# Cardiorespiratory Fitness Estimation Based on Heart Rate and Body Acceleration in Adults With Cardiovascular Risk Factors: Validation Study

**DOI:** 10.2196/35796

**Published:** 2022-10-25

**Authors:** Antti-Pekka E Rissanen, Mirva Rottensteiner, Urho M Kujala, Jari L O Kurkela, Jan Wikgren, Jari A Laukkanen

**Affiliations:** 1 Central Finland Health Care District Jyväskylä Finland; 2 Department of Sports and Exercise Medicine Clinicum University of Helsinki Helsinki Finland; 3 HULA – Helsinki Sports and Exercise Medicine Clinic Foundation for Sports and Exercise Medicine Helsinki Finland; 4 Faculty of Sport and Health Sciences University of Jyväskylä Jyväskylä Finland; 5 Centre for Interdisciplinary Brain Research Department of Psychology University of Jyväskylä Jyväskylä Finland; 6 Institute of Clinical Medicine Department of Medicine University of Eastern Finland Kuopio Finland

**Keywords:** cardiopulmonary exercise test, cardiorespiratory fitness, heart rate variability, hypertension, type 2 diabetes, wearable technology

## Abstract

**Background:**

Cardiorespiratory fitness (CRF) is an independent risk factor for cardiovascular morbidity and mortality. Adding CRF to conventional risk factors (eg, smoking, hypertension, impaired glucose metabolism, and dyslipidemia) improves the prediction of an individual’s risk for adverse health outcomes such as those related to cardiovascular disease. Consequently, it is recommended to determine CRF as part of individualized risk prediction. However, CRF is not determined routinely in everyday clinical practice. Wearable technologies provide a potential strategy to estimate CRF on a daily basis, and such technologies, which provide CRF estimates based on heart rate and body acceleration, have been developed. However, the validity of such technologies in estimating individual CRF in clinically relevant populations is poorly known.

**Objective:**

The objective of this study is to evaluate the validity of a wearable technology, which provides estimated CRF based on heart rate and body acceleration, in working-aged adults with cardiovascular risk factors.

**Methods:**

In total, 74 adults (age range 35-64 years; n=56, 76% were women; mean BMI 28.7, SD 4.6 kg/m^2^) with frequent cardiovascular risk factors (eg, n=64, 86% hypertension; n=18, 24% prediabetes; n=14, 19% type 2 diabetes; and n=51, 69% metabolic syndrome) performed a 30-minute self-paced walk on an indoor track and a cardiopulmonary exercise test on a treadmill. CRF, quantified as peak O_2_ uptake, was both estimated (self-paced walk: a wearable single-lead electrocardiogram device worn to record continuous beat-to-beat R-R intervals and triaxial body acceleration) and measured (cardiopulmonary exercise test: ventilatory gas analysis). The accuracy of the estimated CRF was evaluated against that of the measured CRF.

**Results:**

Measured CRF averaged 30.6 (SD 6.3; range 20.1-49.6) mL/kg/min. In all participants (74/74, 100%), mean difference between estimated and measured CRF was −0.1 mL/kg/min (*P*=.90), mean absolute error was 3.1 mL/kg/min (95% CI 2.6-3.7), mean absolute percentage error was 10.4% (95% CI 8.5-12.5), and intraclass correlation coefficient was 0.88 (95% CI 0.80-0.92). Similar accuracy was observed in various subgroups (sexes, age, BMI categories, hypertension, prediabetes, and metabolic syndrome). However, mean absolute error was 4.2 mL/kg/min (95% CI 2.6-6.1) and mean absolute percentage error was 16.5% (95% CI 8.6-24.4) in the subgroup of patients with type 2 diabetes (14/74, 19%).

**Conclusions:**

The error of the CRF estimate, provided by the wearable technology, was likely below or at least very close to the clinically significant level of 3.5 mL/kg/min in working-aged adults with cardiovascular risk factors, but not in the relatively small subgroup of patients with type 2 diabetes. From a large-scale clinical perspective, the findings suggest that wearable technologies have the potential to estimate individual CRF with acceptable accuracy in clinically relevant populations.

## Introduction

Cardiovascular diseases (CVDs) are a major cause of morbidity, mortality, and economic burden worldwide [1]. In addition to conventional modifiable risk factors for CVD, such as smoking, high blood pressure, impaired glucose metabolism, and dyslipidemia, unambiguous epidemiological evidence shows that cardiorespiratory fitness (CRF) also is an independent and modifiable risk factor for nonfatal CVD events and for cardiovascular and all-cause mortality [2-4]. This is physiologically plausible, as while CRF reflects the integrated capacity of respiratory, cardiovascular, and skeletal muscle systems to take up, transport, and use O_2_ [5], reduced CRF reflects insufficiencies in one or several of these systems. Nonetheless, although adding CRF to conventional risk factors improves the prediction of risk for adverse CVD outcomes [4] and thus provides an opportunity to optimize patient management, it is still the only major CVD risk factor that is not routinely determined in everyday clinical practice [6].

CRF is quantified as an individual’s maximal O_2_ uptake or peak O_2_ uptake (VO_2peak_) and typically measured by ventilatory gas analysis during a cardiopulmonary exercise test (CPET) in clinical practice [7]. CPET requires access of a clinician; equipment; proficiency of clinical personnel conducting and interpreting the test; and to determine CRF, maximal effort of an individual performing the test [7]. As such factors may limit the use of CPET for CRF determination in clinical practice, particularly for large-scale risk prediction in asymptomatic individuals, alternative strategies to estimate CRF as part of routine clinical visits have been developed [6]. For example, several nonexercise CRF prediction equations exist; however, their limited accuracy in estimating CRF at an individual level limits their clinical utility [8]. Submaximal exercise tests, based on linear relationships between O_2_ uptake (VO_2_) and either heart rate (HR) or mechanical workload, are another alternative to estimate CRF [9]. However, their weakness is related to accuracy, confounding factors (eg, medications), interindividual personalization, ceiling effect of the predictive parameter such as HR, and learning effect [9]. To highlight the limitations related to nonexercise and exercise equations, a recent comprehensive analysis of 15 different equations revealed that the accuracy of such equations in estimating CRF is limited from the perspective of individualized clinical decision-making [10]. Consequently, further strategies to estimate CRF with clinically acceptable accuracy are welcome.

Easily available technology provides a strategy to estimate CRF as recent technological advances in wearable devices, such as patches, clothing, and wristband monitors, enable the measurement of HR and multiple other health-related physiological signals in free-living conditions [11]. The validity of several wearable technologies in estimating CRF has been evaluated in healthy young individuals [12-15]. However, although one such study has also included a small number of individuals who are middle-aged and obese [16], the validity of wearable technologies to estimate CRF in clinically relevant populations with CVD risk factors, chronic diseases and medications, and heterogeneous fitness levels is poorly known [15].

In this study, we estimated CRF using a wearable single-lead electrocardiogram (ECG) device. The device can detect atrial fibrillation accurately [17], suggesting that it has clinical applicability within phenomena related to HR and HR variability (HRV). For estimating CRF, the technology of the device relies on HR, HRV, and triaxial body acceleration signals and does not require data from any predetermined protocol, but enables the estimation during self-paced walking performed in free-living conditions [18]. Our aim was to examine the validity of the CRF estimate by comparing it with VO_2peak_ measured directly by CPET in a clinically relevant cohort of working-aged adults with heterogeneous CVD risk factor profiles.

## Methods

### Participants

This validation study was a part of a research collaboration entitled *Heart rate variability analytics to support behavioral interventions for chronic disease prevention and management* (HealthBeat) and conducted between Central Finland Health Care District, University of Jyväskylä, and Firstbeat Technologies Oy in Jyväskylä, Finland. The participants in the HealthBeat study were primarily recruited via web-based advertisements, public advertisements delivered to local noticeboards, and asking the personnel of local health care providers to inform their patients about the study. The inclusion criteria of the study were (1) age between 18 and 64 years, (2) BMI <40 kg/m^2^, (3) either previous evidence of prediabetes (ie, impaired fasting glucose and/or impaired glucose tolerance) or type 2 diabetes diagnosed no more than 5 years ago and/or diagnosed arterial hypertension, and (4) overall physical function not preventing the participant from safely performing the experiments including CPET. The exclusion criteria of the study were anemia, cancer, chronic obstructive pulmonary disease, cerebrovascular disease, chronic atrial fibrillation, clinically significant hypertension-mediated organ damage, diagnosed diabetes-related microvascular disease (ie, nephropathy, neuropathy, and retinopathy), heart failure, insulin use, ischemic heart disease, left bundle branch block, obstructive sleep apnea requiring continuous positive airway pressure treatment, pregnancy or breastfeeding, psychotic disorder or some other unstable psychiatric disorder, secondary hypertension, significant deficit in overall physical function, significant or nonspecified valvular disease, specific medications affecting HR and HRV (β-blockers, serotonin and noradrenaline reuptake inhibitors, and tricyclic antidepressants), substance abuse, symptomatic or unstable asthma, and symptomatic or unstable disorder of the thyroid gland.

For those who were interested in participating in the study, preliminary health screening was conducted by telephone. Then, potentially eligible participants were invited to a preparticipation health screening conducted by a physician with the assistance of a nurse. The preparticipation health screening consisted of a thorough interpretation of an individual’s medical history, clinical status, resting blood pressure, resting ECG, and body mass and height measurements. The antecubital venous blood samples drawn after overnight fasting in an accredited laboratory (FimLab Laboratoriot Oy Ltd, Jyväskylä, Finland) complemented the health screening. The blood sampling included the assessment of blood count, lipid profile, glycemic control, electrolyte balance, and renal function. Frequency, intensity, and duration of both commuting and leisure-time physical activity were obtained as a part of the screening, and total physical activity volume was subsequently expressed as the sum score of metabolic equivalent (MET) hours per day [19] by using the latest available MET values [20]. Overall, the screening focused on evaluating the individual’s signs or symptoms; known cardiovascular, metabolic, or renal disease; current level of physical activity; and desired exercise intensity, as recommended [21].

Altogether, 87 individuals were eligible to participate in the HealthBeat study according to the preparticipation health screening. Of these 87 individuals, planned CPET of 4 (5%) participants was canceled owing to logistic or regulatory circumstances related to the COVID-19 pandemic; 4 (5%) participants withdrew before the planned CPET owing to individual reasons (back pain, lack of time, lower limb pain, and plantar fasciitis); and 1 (1%) participant was excluded after CPET, which unmasked clinical evidence of obstructive coronary artery disease. Therefore, 78 participants who performed CPET for CRF measurement also performed a self-paced walk for CRF estimation. Among the 78 participants, as HR and body acceleration measurements during the self-paced walk were technically unsuccessful in the case of 4 (5%) participants, 74 (95%) participants were eventually included in the final analyses of this study.

### CPET Procedure

To complete CPET, the participants reported to a laboratory for a visit comprising pre-exercise measurements and a graded treadmill walking test. Before the visit, the participants were advised to avoid strenuous physical activity and alcohol use for at least 36 hours and any eating and consumption of coffee, tea, cola, or other stimulative drinks for at least two hours. Body mass and composition were measured using a bioimpedance device (InBody770; InBody Co. Ltd) with the participant in bare feet and light clothing. Body mass and height were used for the calculation of BMI. Waist circumference was measured using stretch-resistant tape at the midpoint between the superior iliac spine and the margin of the lower rib. The circumference was measured to the nearest 0.5 cm and the mean of 2 measurements was calculated. Resting blood pressure was measured with an automated sphygmomanometer device (SunTech Tango M2; SunTech Medical, Inc), and 12-lead ECG (CardioSoft V5.02; GE Medical Systems Information Technologies GmbH) was recorded in the supine position after 5-minute supine rest. Fingertip capillary blood was drawn to measure blood glucose concentration (evercare genius; TaiDoc Technology Corporation) from participants with diabetes to confirm their pre-exercise glucose level being between 5 and 13.9 mmol/L as recommended [22].

The participants performed CPET on a treadmill (JUOKSUMATTO OJK-1; Telineyhtymä) under the supervision of a physician and a nurse. The USAFSAM protocol [23] was used: the test began with 5 minutes of standing at rest, which was followed by 3 minutes of walking at 3.2 km/h (incline 0%), after which the walking speed was set at 5.3 km/h, and the incline of the treadmill was then increased by 5% every 3 minutes until the participant’s volitional task failure. Exercise cessation was followed by 5 minutes of recovery, comprising 1 minute in standing position and subsequent 4 minutes in supine position. During CPET, breath-by-breath inspiratory and expiratory volumes and flows were measured using a low-resistance volume turbine (Triple V, Erich Jaeger), and breath-by-breath inspired and expired gases were sampled continuously at the mouth for the analysis of O_2_ and CO_2_ concentrations (Oxycon Pro Version 5.0; VIASYS Healthcare GmbH). Before each CPET, automatic calibration of the turbine volume transducer and gas analyzer was performed according to the manufacturer’s guidelines. The 12-lead ECG and arterial O_2_ saturation obtained using fingertip pulse oximetry (Nellcor PM10N; Covidien Ilc) were monitored throughout CPET. Systemic arterial blood pressure was measured with the automated sphygmomanometer device during the last 30 seconds of each exercise stage and before anticipated task failure near peak exercise. The rating of perceived exertion at the end of each exercise stage and at peak exercise was obtained (the Borg 6-20 category scale).

VO_2peak_, representing each participant’s directly measured CRF, was determined as the highest value of a 30-second moving averaging VO_2_ interval [24]. The participants’ measured CRF was also characterized as the percentage of predicted VO_2peak_ in relation to Norwegian reference data on VO_2peak_ (mL/kg/min) [25]. As no Finnish reference values exist for treadmill CPET data, the particular data set was used owing to the geographical proximity of Norway to Finland; importantly, considerable differences exist between different reference data sets, and this is partly because of geographical variation [26]. To determine whether the participants achieved VO_2_ plateau, a previously described method to detect each participant’s possible VO_2_ plateau was used [27]: A scatter plot of VO_2_ versus CPET time was first inspected for detecting deviation from linearity, and if evidence of such deviation was observed, a regression line was fitted to the 4 minutes of VO_2_ data preceding the starting point of the deviation. Then, the regression line was extrapolated to the last completed 30 seconds of CPET, and if the difference between this extrapolated VO_2_ value and the participant’s VO_2peak_ was >50% of the slope of the regression line, the participant was concluded to demonstrate VO_2_ plateau.

### Self-paced Walk

To complete a self-paced walk for CRF estimation, the participants reported to an indoor hall for a beforehand scheduled walk after CPET; the median of the gap between CPET and the self-paced walk was 3 days with an IQR of 2 to 7 days. The participants performed a 30-minute self-paced walk on a 200-meter indoor track under the supervision of a physician or nurse. The distance walked in 30 minutes was documented. During the walk, the participants wore a lightweight device (Bodyguard 2; Firstbeat Technologies Oy) attached with 2 skin electrodes on the chest [17] to obtain each participant’s estimated CRF.

### Wearable Device

The wearable device (Bodyguard 2; Firstbeat Technologies Oy) worn during the 30-minute self-paced walk recorded continuous beat-to-beat R-R intervals (ECG sampling frequency: 1000 Hz; R-R interval accuracy: 1 ms) and triaxial body acceleration (movement sampling frequency: 12.5 Hz), and thus provided each participant’s estimated CRF (ie, estimated VO_2peak_ in mL/kg/min). The technology of the device to provide estimated CRF has been developed by Firstbeat Technologies Oy and relies on HR, HRV, and body acceleration signals; the method has been described elsewhere [18]. Although the technology is built on the known, relatively linear relationship between HR and external workload during exercise, it does not require data from any predetermined protocol, but allows CRF estimation from self-paced walking periods performed in free-living conditions. Walking periods providing the most reliable data points and segments for CRF estimation are automatically identified during recording, after which the reliability of the data is automatically evaluated and then used for CRF estimation together with individual background information including at least age, sex, body mass, height, and either age-predicted or known maximal HR. In this study, the background information acquired during the CPET visit was used to obtain estimated CRF, and thus included age, sex, body mass, height, and known maximal HR. The exact algorithm behind the CRF estimation technology is proprietary; thus, it is inaccessible and not presented here.

### Statistical Analysis

Descriptive statistics were used to characterize the participants. Mean difference between the estimated and measured CRF was quantified (difference=estimated CRF−measured CRF) and evaluated using paired-samples 2-tailed *t* test. Mean absolute error (MAE; absolute error=|estimated CRF–measured CRF|) and mean absolute percentage error (MAPE; absolute percentage error=[|estimated CRF–measured CRF|]/measured CRF×100%) of the estimated CRF were calculated to describe the magnitude of error for individual-level estimation [28]. Intraclass correlation coefficients (ICCs) were determined to test the overall concordance between estimated and measured CRF [29]. Bland-Altman plot complemented the validity analyses to visually demonstrate the level of agreement between estimated and measured CRF with 95% limits of agreement across the whole range of CRF levels [30]. Shapiro-Wilk test (in case of a sample size <50 participants) and Kolmogorov-Smirnov (in case of a sample size ≥50 participants) test were used to test the normality of the data. In case of absolute and absolute percentage errors, nonnormally distributed subgroup-specific data were bootstrapped (×10,000) to present the data with 95% CIs. Data are presented as mean (SD) or mean (95% CI) for normally distributed continuous variables, median (IQR) for nonnormally distributed continuous variables, and n (%) for categorical variables. Statistical analyses were performed using IBM SPSS Statistics 26.0 (IBM), and the statistical significance was set at *P*<.05.

### Ethics Approval

The HealthBeat study was conducted according to the guidelines of the Declaration of Helsinki and approved by the ethics committee of the Central Finland Hospital District, Jyväskylä, Finland (Dnro 23U/2018). All participants provided their oral and written informed consent before their participation.

## Results

### Participants

The participants were Finns. Table 1 presents the participants’ descriptive characteristics. To complement the data in Table 1, 5% (4/74) of the participants had first-degree atrioventricular block, but resting 12-lead ECG did not reveal any significant rhythm or conduction abnormalities. Table 2 presents the relevant cardiometabolic risk factors and medications used by the participants. Overall, the participants’ CVD risk factor profiles were heterogeneous, as shown in Tables 1 and 2.

**Table 1:** Descriptive characteristics (N=74)^a^.

Characteristics		Data	Range
**Sex,** **n (%)**			
	Female	56 (76)	N/A^b^
	Male	18 (24)	N/A
Age (years), median (IQR)		55.8 (49.9-59.5)	34.8-64.5
Physical activity (MET^c^ hours per day), median (IQR)		2.6 (1.3-4.9)	0.04-15.4
**Body size and composition**			
	Body mass (kg), mean (SD)	82 (16.7)	53.6-135.8
	Height (cm), median (IQR)	165 (162-175)	152-191
	BMI (kg/m^2^)	28.7 (4.6)	21.9-39.9
	Fat-free mass (kg), median (IQR)	50.8 (46.7-61.1)	35.9-84.7
	Fat percentage (%), mean (SD)	33 (9)	12-51
	Fat mass (kg), mean (SD)	27.6 (10.5)	9.5-54.7
	Waist circumference (cm), mean (SD)	98 (13)	74-132
**Blood samples**			
	Hemoglobin (g/L), mean (SD)	143 (10)	123-167
	Total cholesterol (mmol/L), mean (SD)	4.9 (0.9)	2.8-7
	LDL^d^ cholesterol (mmol/L), mean (SD)	3.1 (0.9)	1.4-5.1
	HDL^e^ cholesterol (mmol/L), mean (SD)	1.5 (0.4)	0.9-2.4
	Triglycerides (mmol/L), median (IQR)	1.1 (0.8-1.8)	0.4-4
	Fasting glucose (mmol/L), median (IQR)	5.7 (5.2-6.2)	4.6-7.8
	HbA_1__c_^f^ (mmol/mol), median (IQR)	38 (35-40)	31-50
	Estimated GFR^g^ (mL/min/1.73 m^2^), mean (SD)	84 (13)	56-125
**Resting hemodynamics**			
	Sinus rhythm, n (%)	74 (100)	N/A
	Heart rate (bpm^h^), median (IQR)	67 (61-76)	48-105
	Systolic blood pressure (mm Hg), mean (SD)	135 (13)	106-178
	Diastolic blood pressure (mm Hg), mean (SD)	83 (7)	64-98

^a^Data are presented as mean (SD) for normally distributed continuous variables, median (IQR) for nonnormally distributed continuous variables, and n (%) for categorical variables.

^b^N/A: not applicable.

^c^MET: metabolic equivalent.

^d^LDL: low-density lipoprotein.

^e^HDL: high-density lipoprotein.

^f^HbA_1c_: glycosylated hemoglobin A_1c_.

^g^GFR: glomerular filtration ratio.

^h^bpm: beats per minute.

**Table 2:** Cardiometabolic risk factors and medications (N=74).

		Data, n (%)
Arterial hypertension		64 (86)
Prediabetes^a^		18 (24)
Type 2 diabetes		14 (19)
Metabolic syndrome^b^		51 (69)
**Smoking**		
	Yes	4 (5)
	No	70 (95)
**Family history of premature heart disease^c^**		
	Yes	21 (28)
	No	47 (64)
	Do not know	6 (8)
**Medication**		
	ACE^d^ or ARB^e^	55 (74)
	Calcium channel blockers	18 (24)
	Diuretics	11 (15)
	Statins	14 (19)
	Tablet treatment for diabetes	12 (16)

^a^Evidence of impaired fasting glucose (6.1-6.9 mmol/L) previously or during this study and previous evidence of impaired glucose tolerance, but no type 2 diabetes.

^b^As defined by the International Diabetes Federation [31].

^c^Sudden cardiac death, angina pectoris, or coronary artery disease at young age (ie, men aged <55 years and women aged <65 years) in first-degree relatives.

^d^ACE: angiotensin-converting enzyme inhibitor.

^e^ARB: angiotensin receptor blocker.

### CPET and Self-paced Walk

Table 3 presents CPET and self-paced walk data. On the basis of respiratory exchange ratio, rating of perceived exertion, and percentage of age-predicted maximal HR, the participants performed their maximal effort during CPET, whereas only 36% (27/74) of the participants achieved VO_2_ plateau along with previous observations [25]. Measured VO_2peak_ ranged from 20.1 to 49.6 mL/kg/min, and the participants represented different CRF categories as shown in Table 3.

**Table 3:** Cardiopulmonary exercise test and self-paced walk (N=74)^a^.

Methods					Data
**Cardiopulmonary exercise test**					
	Exercise time (minutes), mean (95% CI)			15.6 (15-16.2)	
	Measured VO_2peak_^b^ (L/min), median (IQR)			2.3 (2-2.8)	
	Measured VO_2peak_ (mL/kg/min), mean (95% CI)			30.6 (29.2-32.1)	
	Measured VO_2peak_ (mL/kg FFM^c^/min), mean (95% CI)			45.6 (44.3-46.9)	
	Measured VO_2peak_ (percentage of predicted VO_2peak_)^d^, mean (95% CI)			94 (90-98)	
	Achieved VO_2_^e^ plateau, n (%)			27 (36)	
	Maximal V_E_^f^ (L/min), median (IQR)			88 (75-110)	
	Maximal RER^g^, mean (95% CI)			1.16 (1.15-1.18)	
	Maximal RPE^h^, median (IQR)			19 (17-19)	
	SpO_2_^i^ at peak exercise (%), mean (95% CI)			95 (95-96)	
	Maximal HR^j^ (bpm^k^), mean (95% CI)			172 (169-175)	
	Maximal HR (percentage of age-predicted maximal HR)^l^, mean (95% CI)			103 (102-105)	
	Maximal systolic blood pressure (mm Hg), mean (95% CI)			216 (210-221)	
	Maximal diastolic blood pressure (mm Hg), mean (95% CI)			94 (91-96)	
	**CRF^m^ category (percentage of predicted VO_2peak_)^d^, n (%)**				
		<80	15 (20)		
		80-99	38 (51)		
		100-120	16 (22)		
		>120	5 (7)		
**Self-paced walk, mean (95% CI)**					
	Walking distance (m)			3174 (3114-3235)	
	Estimated VO_2peak_ (mL/kg/min)			30.6 (29.2-32)	

^a^Data are presented as mean (95% CI) for normally distributed continuous variables, median (IQR) for nonnormally distributed continuous variables, and n (%) for categorical variables.

^b^VO_2peak_: peak O_2_ uptake.

^c^FFM: fat-free mass.

^d^Predicted VO_2peak_ based on Edvardsen et al [25].

^e^VO_2_: O_2_ uptake.

^f^V_E_: minute ventilation.

^g^RER: respiratory exchange ratio.

^h^RPE: rating of perceived exertion.

^i^SpO_2_: arterial O_2_ saturation.

^j^HR: heart rate.

^k^bpm: beats per minute.

^l^Age-predicted maximal HR=220–age.

^m^CRF: cardiorespiratory fitness.

### Validity of Estimated CRF in All Participants

**Figure 1:**
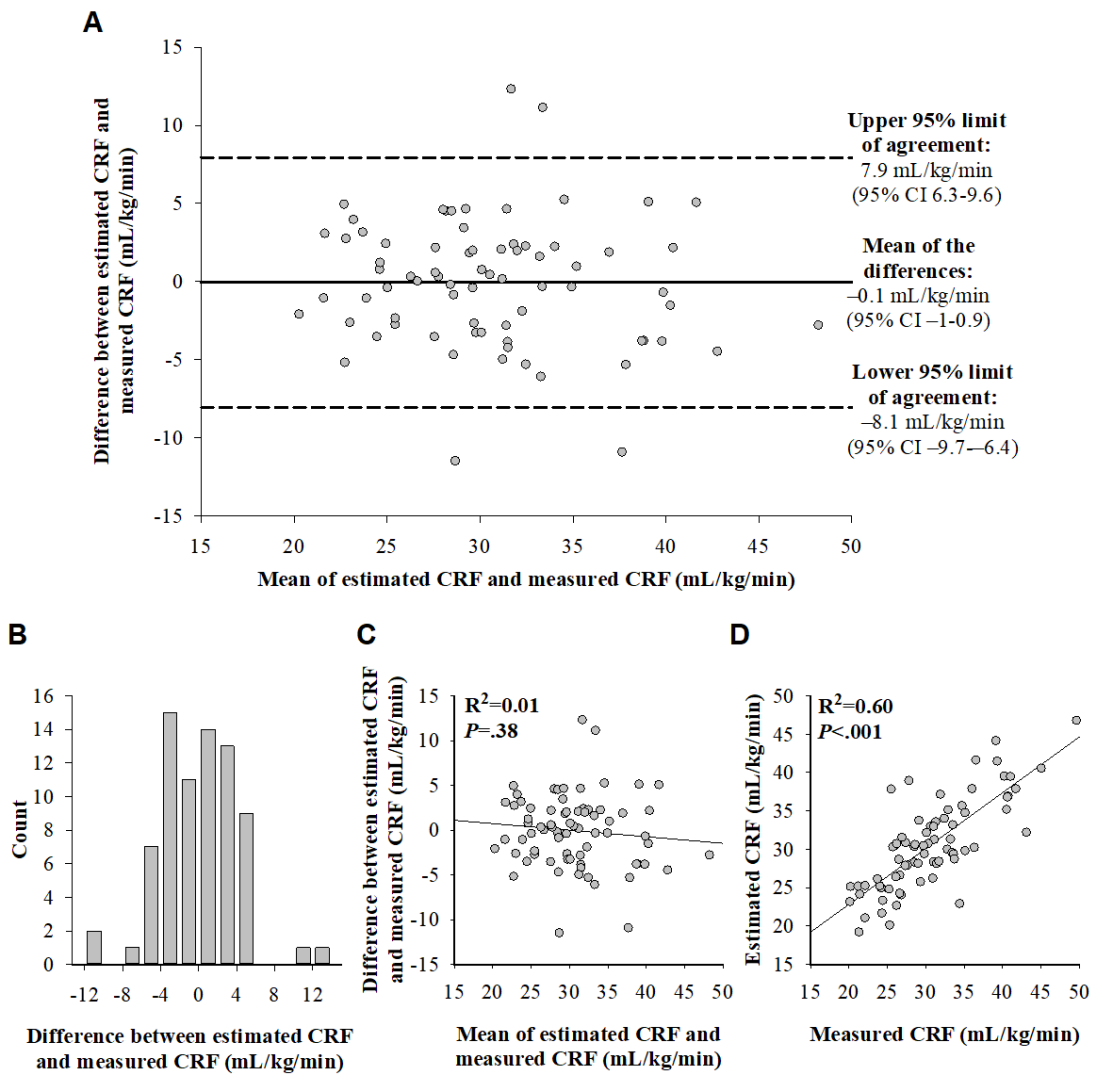
(A) Bland-Altman plot for agreement between estimated cardiorespiratory fitness (CRF; ie, peak O_2_ uptake in mL/kg/min, estimated based on a 30-minute self-paced walk) and measured CRF (ie, peak O_2_ uptake in mL/kg/min, measured using a cardiopulmonary exercise test) in all participants (74/74, 100%). The solid horizontal line represents the mean of the differences between the methods, and the dashed lines represent the upper and lower 95% limits of agreement. (B) Distribution histogram of the differences between estimated and measured CRF, which are normally distributed (Kolmogorov-Smirnov test, *P*=.20; Shapiro-Wilk test, *P*=.07). (C) Scatter plot with regression fit of the differences between estimated and measured CRF versus the means of the estimated and measured CRF. (D) Scatter plot with regression fit of estimated CRF versus measured CRF. CRF: cardiorespiratory fitness.

The pooled analysis of all 74 participants revealed that the mean difference between estimated and measured CRF was minimal (−0.1 mL/kg/min; *P*=.90; Figure 1; Table 4). MAE was 3.1 mL/kg/min and MAPE was 10.4% (Table 4). In addition, ICC (*r*=0.88; 95% CI 0.80-0.92) demonstrated good concordance between the 2 methods (Table 4). According to the Bland-Altman plot and its complementary graphs (Figure 1), the level of agreement between estimated and measured CRF was similar across the whole range of CRF levels; however, 5% (4/74) of the participants had their between-method difference beyond the 95% limits of agreement. A detailed inspection of the characteristics of that 5% (4/74) of the participants did not reveal any specific explanation for such exaggerated differences (Multimedia Appendix 1 [25,31]).

**Table 4:** Mean differences between estimated CRF^a^ (ie, peak O_2_ uptake in mL/kg/min, estimated based on a 30-minute self-paced walk) and measured CRF (ie, peak O_2_ uptake in mL/kg/min, measured through a cardiopulmonary exercise test), mean absolute and mean absolute percentage errors of estimated CRF, and ICCs^b^ between estimated and measured CRF for all participants and subgroups (N=74)^c^.

		Participants, n (%)	Paired-samples *t* test^d^		Errors, mean (95% CI)			ICC, *r* (95% CI)
			Difference (mL/kg/min), mean (95% CI)	*P* value	Absolute error (mL/kg/min)	Absolute percentage error (%)		
All		74 (100)	−0.1 (−1 to 0.9)	.90	3.1 (2.6 to 3.7)^e^	10.4 (8.5 to 12.5)^e^	0.88 (0.80 to 0.92)	
**Sex**								
	Female	56 (76)	−0.03 (−1.1 to 1.1)	.96	3 (2.3 to 3.8)	10.5 (8 to 13.1)	0.85 (0.75 to 0.91)	
	Male	18 (24)	−0.2 (−2.2 to 1.8)	.87	3.4 (2.5 to 4.4)	10.1 (7.2 to 13)	0.87 (0.66 to 0.95)	
**Age**								
	Below median (<55.8 years)	37 (50)	−0.7 (−2.2 to 0.7)	.30	3.4 (2.6 to 4.3)^e^	10.5 (8 to 13.5)^e^	0.85 (0.71 to 0.92)	
	Above median (>55.8 years)	37 (50)	0.6 (−0.7 to 1.9)	.33	2.9 (2.1 to 3.8)^e^	10.4 (7.7 to 13.3)^e^	0.88 (0.76 to 0.94)	
**BMI (kg/m^2^)**								
	<25	18 (24)	−1.4 (−3.7 to 1)	.23	3.7 (2.4 to 5.2)^e^	10.5 (6.9 to 14.6)^e^	0.79 (0.44 to 0.92)	
	25-29.99	30 (41)	0.5 (−1.1 to 2.1)	.53	3.2 (2.3 to 4.3)^e^	10.4 (7.1 to 14.4)^e^	0.82 (0.62 to 0.91)	
	≥30	26 (35)	0.2 (−1.1 to 1.5)	.74	2.6 (1.9 to 3.3)	10.4 (7.6 to 13.2)	0.81 (0.57 to 0.91)	
Arterial hypertension		64 (86)	−0.6 (−1.5 to 0.4)	.24	3 (2.4 to 3.6)^e^	9.7 (8 to 11.6)^e^	0.88 (0.80 to 0.93)	
**Glucose metabolism**								
	Normal	42 (57)	−0.7 (−1.7 to 0.4)	.21	2.8 (2.1 to 3.4)^e^	8.5 (6.7 to 10.3)	0.91 (0.84 to 0.95)	
	Prediabetes^f^	18 (24)	−0.4 (−2.5 to 1.6)	.66	3.2 (2.1 to 4.5)^e^	10.2 (7.1 to 14)^e^	0.88 (0.66 to 0.95)	
	Type 2 diabetes	14 (19)	2.3 (−0.7 to 5.2)	.12	4.2 (2.6 to 6.1)^e^	16.5 (8.6 to 24.4)	0.66 (0.03 to 0.89)	
Metabolic syndrome^g^		51 (69)	0.7 (−0.4 to 1.7)	.22	3.0 (2.3 to 3.7)	10.8 (8.2 to 13.3)	0.82 (0.69 to 0.90)	

^a^CRF: cardiorespiratory fitness.

^b^ICC: intraclass correlation coefficient.

^c^Data are presented as mean (95% CI) for the differences and errors and *r* (95% CI) for ICC.

^d^2-tailed.

^e^Bootstrapped (×10,000) owing to originally nonnormally distributed data.

^f^Evidence of impaired fasting glucose (6.1-6.9 mmol/L) previously or during this study and previous evidence of impaired glucose tolerance, but no type 2 diabetes.

^g^As defined by the International Diabetes Federation [31].

### Validity of Estimated CRF in Subgroups

Data related to the validity of estimated CRF in different subgroups are presented in Table 4. The data show that the CRF estimation method was likely to provide similar accuracy in women and men and across age and BMI categories, when comparing with the data on all participants (Table 4). This was also evident in the participants with hypertension, normal glucose metabolism, prediabetes, and metabolic syndrome (Table 4). In contrast, the participants with type 2 diabetes demonstrated lower estimation accuracy than other subgroups; for example, MAPE was 16.5% in those with type 2 diabetes (Table 4). The accuracy of the CRF estimation method was equally good in 36% (27/74) of the participants who achieved VO_2_ plateau at the end of CPET and in 64% (47/74) of the participants who did not achieve it (eg, MAE was 3.3 mL/kg/min, 95% CI 2.3-4.4 and 3 mL/kg/min, 95% CI 2.4-3.8, respectively; MAPE was 10.4%, 95% CI 7.2-13.9 and 10.4%, 95% CI 8.2-13, respectively).

## Discussion

### Principal Findings

Wearable technology provides a strategy to estimate CRF as part of routine clinical practice. In this study, we estimated the CRF of working-aged adults with heterogeneous CVD risk factor profiles with a technology that uses wearable device data on HR, HRV, and body acceleration monitored during self-paced walking. We tested the validity of the technology by comparing the participants’ estimated CRF with their CRF measured directly by ventilatory gas analysis during CPET. For all participants (74/74, 100%), the mean difference between estimated and measured CRF was −0.1 mL/kg/min, MAE was 3.1 mL/kg/min, MAPE was 10.4%, and average ICC was 0.88, reflecting high accuracy of the examined method to estimate CRF. In subgroup analyses, similar accuracy of the CRF estimation method was observed in both sexes, different age and BMI categories, patients with hypertension, patients with prediabetes, and patients with metabolic syndrome. However, the technology did not provide equally accurate CRF estimation in the small subgroup of patients with type 2 diabetes (14/74, 19%), in whom MAE and MAPE were 4.2 mL/kg/min and 16.5%, respectively.

### Comparison With Previous Studies

CRF, quantified as an individual’s maximal VO_2_ or VO_2peak_, reflects the integrated capacity of the respiratory, cardiovascular, and skeletal muscle systems to take up, transport, and use O_2_ [5], and thus it has normal physiological variation. Studies that have examined the test-retest repeatability of CPET parameters in healthy populations have observed the coefficient of variation of directly measured VO_2peak_ to be approximately 5% [32,33]. Such a level of test-retest repeatability is not attained in patient populations. The coefficient of variation of directly measured VO_2peak_ has varied between 6% and 9% in various patient populations such as patients with chronic obstructive pulmonary disease [34], heart failure [34,35], peripheral arterial disease [36], pulmonary arterial hypertension [37], or severe mitral valve disease [34]. In light of these findings, it may be postulated that the MAPE of the CRF estimation method could have ideally been between 5% and 9% in this study, which included a patient cohort with frequent cardiovascular risk factors and medications. Thus, as the MAPE of estimated CRF varied between 8.5% and 10.8% in both the pooled cohort and all subgroups in this study, except for the patients with type 2 diabetes, the accuracy of the method to estimate CRF can be considered to be acceptable in terms of the inevitable physiological variation of CRF.

Wearable technology provides an approach to estimate CRF for routine individualized risk prediction in everyday clinical practice. The validity of several wearable technologies that provide CRF estimations has been studied [12-16]. Similar to the CRF estimation method examined in this study, most of the technologies have used HR and body acceleration data [12,14-16], whereas some technologies combine HR and body acceleration data with data from respiratory bands [13]. MAPE of such estimates has ranged from 8% to 10.2% [14,15]. From the clinical perspective, it is important to note that the participants in most of those studies were healthy and relatively young and fit [12-15]; however, one such study has also included 9 individuals aged >50 years and 7 individuals who were obese [16]. In consequence, the need for validation studies including clinically relevant populations (eg, older people, individuals who are obese, and individuals with chronic diseases) has been highlighted [12,14,15]. In this regard, it is noteworthy that, when compared with the previously reported accuracies of the other technologies, the accuracy of the CRF estimation method examined in this study was similar and particularly did so in the clinically relevant cohort with heterogeneous and comprehensively reported CVD risk profiles.

The accuracy of the CRF estimation method was lower for the participants with type 2 diabetes (14/74, 19%) than for the pooled study cohort or other subgroups. For instance, MAE was 4.2 mL/kg/min and MAPE was 16.5% for the participants with diabetes. Patients with diabetes are prone to cardiac autonomic neuropathy, the signs and symptoms of which include reduced HRV, resting tachycardia, abnormal blood pressure regulation, orthostatic hypotension, orthostatic tachycardia or bradycardia, chronotropic incompetence, and exercise intolerance [38]. In addition, exaggerated HRV complexity during CPET has been observed in working-aged adults with well-controlled type 1 diabetes [39]. Although the prevalence of diabetes-related cardiac autonomic neuropathy increases with diabetes duration and may be evident in 60% of patients with type 2 diabetes after 15 years, cardiac autonomic neuropathy may also be asymptomatic and manifest only as reduced HRV [38]. Thus, it may be that the reduced accuracy of the CRF estimation method in the type 2 diabetes subgroup was owing to early diabetes-related disturbances in cardiac autonomic modulation, although the participants with type 2 diabetes had good glycemic control in terms of glycosylated hemoglobin A_1c_, short diabetes duration (from 0.5 to 4.4 years), and no previous evidence of autonomic neuropathy. Importantly, the accuracy of the CRF estimation method was not reduced in the subgroups with prediabetes or metabolic syndrome. This suggests that the method provides an accurate estimation of CRF in the 2 clinically relevant patient groups; however, this may not be the case for patients with both metabolic syndrome and type 2 diabetes. Overall, as the subgroup with type 2 diabetes included only 19% (14/74) of the participants, future validation studies including large number of patients with diabetes are warranted.

The findings of this study have relevant clinical applicability. As epidemiological evidence shows that CRF independently predicts incidence and mortality of not only CVD but also respiratory diseases and cancer and all-cause mortality [2-4], determining CRF as a vital sign in routine clinical practice as recommended may lead to several health benefits [6]. For example, identifying individuals with low CRF and thus increased risk for adverse health outcomes may guide health care providers to target more intensive preventive interventions at such individuals. CRF can be used as a medium for facilitating discussions about individual health concerns and lifestyle modification, and determined CRF can also be added to classic risk algorithms to improve the accuracy of individual risk prediction [6,40]. For such daily clinical purposes, the feasibility to use CPET may be limited by requirements related to costs, expertise, resources, and effort dependency [7]. In addition, the feasibility to use exercise-based prediction equations for individualized clinical decision-making is limited by the accuracy of such equations. This was recently demonstrated by Peterman et al [10], who reported limited accuracy levels of 2 nonexercise (SE of estimate [SEE] 4.9 mL/kg/min), 3 submaximal exercise (SEE 7-9.1 mL/kg/min), and 10 maximal exercise equations (SEE 3.6-5.6 mL/kg/min; except for 1 equation with SEE of 2.5 mL/kg/min). Regarding the CRF estimation method examined in this study, MAE was 3.1 mL/kg/min in the pooled study cohort and 2.6 to 3.7 mL/kg/min in each subgroup, except for the participants with type 2 diabetes. Thus, the overall level of accuracy was higher than the recently reported levels of the prediction equations [10]. In addition, although approximately one-third (27/74, 36%) of participants had their absolute error >1 MET (ie, >3.5 mL/kg/min), MAE of 3.1 mL/kg/min was <1 MET, which is noteworthy because even +1 or –1 MET translates into prognostically significant CRF deviation [6]. Furthermore, the Bland-Altman plot and its complementary analyses (Figure 1) demonstrate that the level of accuracy was similar across the whole range of CRF levels. In summary, the accuracy of the CRF estimation method may be considered as likely sufficient for individualized clinical decision-making, irrespective of the individual’s CRF level.

### Strengths and Limitations

The main strength and the main novelty of this study reside in the characteristics of the participants: The working-aged adults comprised a clinically relevant cohort with frequent cardiovascular risk factors (eg, hypertension and impaired glucose metabolism) and common medications (eg, angiotensin-converting enzyme inhibitors, angiotensin receptor blockers, statins, and metformin). The need for strategies to estimate CRF with clinically acceptable accuracy in such individuals has been highlighted [6,12,14,15]. The cohort size was also relatively large compared with previous similar validation studies examining healthy individuals [12-16]; however, the sex distribution was not optimally balanced (women: 56/74, 76% and men: 18/74, 24%). An important limitation of this study is that CRF was estimated based on a standard 30-minute self-paced walk. Thus, the validity of the CRF estimation method remains to be tested under completely free-living conditions. In addition, the risk of recruitment bias may not be optimally avoided, as the median volume of total physical activity of the participants was 2.6 MET hours per day, which approximately corresponds, for example, to 30 minutes of moderate-intensity aerobic activity per day [41]. This may reflect the tendency for physically active individuals to volunteer for this type of study that includes exercise provocations. However, the average CRF of the participants was 94% of predicted, the participants represented a wide spectrum of different CRF categories, and importantly, the accuracy level of the CRF estimation method was similar across the measured VO_2peak_ range of 20.1 to 49.6 mL/kg/min. Thus, the findings and conclusions of this study can be generalized to working-aged adults with frequent cardiovascular risk factors and VO_2peak_ >20 mL/kg/min but without the exclusion criteria of this study.

### Conclusions

We estimated the CRF of 74 working-aged adults with heterogeneous CVD risk factor profiles with a technology that uses wearable device data on HR, HRV, and body acceleration monitored during self-paced walking. After comparing the participants’ estimated CRF with their directly measured CRF, we conclude that, in populations comparable with the cohort examined in this study, the error of the CRF estimate is likely below or at least very close to 1 MET. This is relevant because even +1 or –1 MET translates into prognostically significant CRF deviation [6]. Such accuracy was observed in the pooled study cohort and various subgroups including both sexes, different age and BMI categories, patients with hypertension, patients with prediabetes, and patients with metabolic syndrome, but not in a small subgroup of patients with type 2 diabetes (14/74, 19%). Future studies are warranted to examine the validity of the method in large type 2 diabetes cohorts, under completely uncontrolled free-living conditions, and in test-retest and longitudinal settings to evaluate whether the method can be used for clinical follow-up purposes.

From a large-scale clinical perspective, this study suggests that wearable technologies may have the potential to estimate individual CRF with acceptable accuracy in clinically relevant populations and thus aid in improving the prediction of individual risk for adverse health outcomes such as adverse CVD events.

## Appendix

Multimedia Appendix 1 Characteristics of the 5% (4/74) of the participants (1-4), in whom the difference between estimated and measured cardiorespiratory fitness fell beyond the 95% limits of agreement, as shown in Figure 1.

## Abbreviations

CPET: cardiopulmonary exercise test
CRF: cardiorespiratory fitness
CVD: cardiovascular disease
ECG: electrocardiogram
HealthBeat: Heart rate variability analytics to support behavioral interventions for chronic disease prevention and management
HR: heart rate
HRV: heart rate variability
ICC: intraclass correlation coefficient
MAE: mean absolute error
MAPE: mean absolute percentage error
MET: metabolic equivalent
SEE: SE of estimate
VO_2_: O_2_ uptake
VO_2peak_: peak O_2_ uptake

## Data Availability

The data underlying the reported conclusions will be shared upon request to the corresponding author.
