# Supplementary material for: Cardiorespiratory Fitness Estimation Based on Heart Rate and Body Acceleration in Adults With Cardiovascular Risk Factors: Validation Study
Source: JMIR Cardio. 2022 Oct 25;6(2):e35796. doi: 10.2196/35796 (PMC9644248; doi:10.2196/35796)
Supplement: Multimedia Appendix 1 [file cardio_v6i2e35796_app1.docx]

**Supplementary Table 1.** Characteristics of the 5% (4/74) of the participants (1-4), in whom the difference between estimated and measured cardiorespiratory fitness fell beyond the 95% limits of agreement, as shown in Figure 1.

|  | **1** | **2** | **3** | **4** |
| --- | --- | --- | --- | --- |
| Sex | female | female | female | female |
| Age | 51 | 57 | 61 | 52 |
| Physical activity  (MET hours per day) | 7.6 | 0.8 | 6.1 | 5.9 |
| Body size and composition |  |  |  |  |
| Body mass (kg) | 59.8 | 71.7 | 59.4 | 79.6 |
| Height (cm) | 158 | 161 | 158 | 172.5 |
| Body mass index (kg/m^2^) | 24 | 27.7 | 23.8 | 26.8 |
| Fat percentage (%) | 26.6 | 33.6 | 34.2 | 35.0 |
| Waist circumference (cm) | 76.5 | 100.0 | 88.0 | 92.0 |
| Arterial hypertension | yes | yes | yes | no |
| Type 2 diabetes | no | yes | no | yes |
| HbA_1c_ (mmol/mol) | 35 | 40 | 37 | 48 |
| Other diagnoses | no | stable hypothyroidism | stable hypothyroidism | no |
| Metabolic syndrome ^a^ | no | yes | no | yes |
| Medication | losartan | levothyroxine, losartan, metformin | amlodipin, hydrochlorothiazide, levothyroxine, ramipril | melatonin, metformin, rosuvastatin |
| Smoking | no | no | no | no |
| Cardiopulmonary exercise test |  |  |  |  |
| Exercise time (min) | 21 | 14.2 | 13.2 | 15 |
| Measured VO_2peak_ (L/min) | 2.6 | 2.0 | 2.1 | 2.0 |
| Measured VO_2peak_  (mL/kg/min) | 43.1 | 27.8 | 34.4 | 25.5 |
| Measured VO_2peak_  (% of predicted VO_2peak_) ^b^ | 135 | 93 | 120 | 81 |
| Maximal RER | 1.16 | 1.13 | 1.06 | 1.12 |
| Maximal HR (bpm) | 169 | 196 | 181 | 164 |
| Maximal HR (% of age-predicted maximal HR) ^c^ | 100 | 120 | 114 | 98 |
| Self-paced walk |  |  |  |  |
| Walking distance (m) | 3305 | 2940 | 3325 | 3095 |
| Estimated VO_2peak_  (mL/kg/min) | 32.2 | 38.9 | 22.9 | 37.8 |
| CRF errors |  |  |  |  |
| Absolute error  (mL/kg/min) | 10.9 | 11.2 | 11.5 | 12.3 |
| Absolute percentage error (%) | 25.4 | 40.2 | 33.4 | 48.3 |

^a^ As defined by the International Diabetes Federation [41].

^b^ Predicted V̇O_2peak_ based on Edvardsen et al. [25].

^c^ Age-predicted maximal HR = 220 – age.

CRF, cardiorespiratory fitness; HbA_1c_, glycosylated hemoglobin A_1c_; HR, heart rate; MET, metabolic equivalent; RER, respiratory exchange ratio; VO_2peak_, peak O_2_ uptake.
